# Phospholipase A2 and Systemic‐Immune Inflammation Index as Early Predictors of Neurotoxicity Induced by Acute Glufosinate Ammonium Poisoning: A Population‐Based Case–Control Analysis

**DOI:** 10.1155/emmi/9034089

**Published:** 2026-01-03

**Authors:** Xiang Xue, Xinyao Wu, Zhaorui Sun, Shinan Nie, Changbao Huang

**Affiliations:** ^1^ Department of Emergency Medicine, The First Affiliated Hospital of Wannan Medical College (Yijishan Hospital of Wannan Medical College), Wuhu, China, wnmc.edu.cn; ^2^ Department of Emergency Medicine, Jinling Hospital, Affiliated Hospital of Medical School, Nanjing University, Nanjing, 210002, China, nju.edu.cn; ^3^ Department of Emergency Medicine, Jinling Clinical Medical College, Nanjing University of Chinese Medicine, Nanjing, 210002, China, njucm.edu.cn

**Keywords:** glufosinate ammonium, neurotoxicity, phospholipase A2, population-based, Systemic-Immune Inflammation Index

## Abstract

**Objective:**

This study aimed to investigate the predictive value of the Systemic‐Immune Inflammation Index (SII) and lipoprotein‐associated phospholipase A2 (Lp‐PLA2) in the early detection of glufosinate ammonium (GA) poisoning‐induced neurotoxicity.

**Methods:**

A retrospective case–control analysis of patients with acute oral GA poisoning was conducted from January 2021 to August 2024. GA poisoning patients who developed neurotoxicity were identified as the case group. The control group was matched 1:2 with the case group on the year of age interval in GA patients without neurotoxicity. Univariate and multiple logistic regression analyses were performed to explore the independent risk of neurotoxicity induced by GA poisoning. Receiver operator characteristic (ROC) curve and area under the curve (AUC) were performed to evaluate the predictive value of SII, Lp‐PLA2, and combination of both in GA poisoning patients associated with neurotoxicity.

**Results:**

A cohort of 82 patients experiencing neurotoxicity due to GA poisoning was identified, alongside a control group of 164 individuals who did not exhibit neurotoxic symptoms. The levels of SII and Lp‐PLA2 were higher among the case group compared with the control group. After controlling for plasma GA concentration, lactate, neutrophil‐to‐lymphocyte ratio, and serum ammonia, the results of the multiple logistic regression analysis indicated that the case group was more likely to exhibit elevated levels of the SII (OR = 1.010, 95% CI: 1.004, 1.015, *p* < 0.001) and Lp‐PLA2 (OR = 1.049, 95% CI: 1.032, 1.065, *p* < 0.001). Furthermore, the areas under the ROC curve of SII, Lp‐PLA2, and combination of both were 0.781 (95% CI: 0.717, 0.845, *p* < 0.001), 0.880 (95% CI: 0.838, 0.923, *p* < 0.001), and 0.931 (95% CI: 0.901, 0.961, *p* < 0.001), respectively.

**Conclusions:**

The study concluded that SII, Lp‐PLA2, and their combination could serve as predictive biomarkers for assessing the neurotoxicity associated with glufosinate ammonium poisoning.

## 1. Introduction

Glufosinate ammonium (GA), a commonly utilized nonselective herbicide in contemporary agricultural practices, presents several advantages over traditional herbicides, including user‐friendly application, extensive efficacy, and prolonged residual activity [1, 2]. The increased agricultural application of GA has been associated with a heightened incidence of intentional poisonings, leading to severe toxicity that impacts the neurological system, reproductive system, and respiratory system [3–5]. Research has indicated that approximately 70% of individuals experiencing GA poisoning exhibit delayed neurotoxicity, which is characterized by symptoms including memory impairment, coma, convulsions, and seizures [6–8]. Forecasting neurotoxicity in asymptomatic patients within the emergency department presents significant challenges, requiring vigilant observation over several days following ingestion, irrespective of initial clinical manifestations. Research indicates that serum and cerebrospinal fluid (CSF) ammonia levels may function as prognostic indicators of neurotoxic effects in cases of GA poisoning [9, 10]. Due to the low sensitivity and specificity of serum ammonia tests and the lengthy process of detecting ammonia in CSF, serum ammonia is not practical as a prognostic marker for GA‐induced neurotoxicity. This study aimed to find a more cost‐effective and accessible prognostic indicator.

Lipoprotein‐associated phospholipase A2 (Lp‐PLA2) is a critical enzyme involved in vascular inflammation and atherogenesis. It is primarily localized within macrophages and other proinflammatory cells present in atherosclerotic lesions, demonstrating a high affinity for low‐density lipoprotein. This interaction exacerbates inflammatory responses and facilitates disease progression [11, 12]. The role of Lp‐PLA2 in inflammatory processes suggests its potential involvement in the neurological effects associated with GA poisoning. Furthermore, the Systemic Immune‐Inflammation Index (SII), derived from peripheral blood cell counts, has emerged as a novel marker for evaluating systemic inflammation and immune status. Empirical evidence indicates that SII possesses prognostic value in various conditions, including cancer and cardiovascular diseases [13, 14]. Nonetheless, its efficacy in predicting brain damage resulting from GA poisoning remains to be elucidated.

This study aimed to elucidate the relationship between Lp‐PLA2, SII, and neurotoxicity resulting from exposure to GA. Considering the age‐related rise in the incidence of nerve damage due to GA toxicity, the analysis was further stratified by age to enhance its precision.

## 2. Materials and Methods

### 2.1. Selection of Cases and Controls

This retrospective study investigates patients aged 18 years and older who were diagnosed with acute GA poisoning and subsequently admitted to the emergency department at Jinling Hospital, affiliated with the Medical School of Nanjing University, during the period from January 2021 to August 2024. Given that this research employs a case–control study design and the exposure factor being examined is a categorical variable, the sample size for the control group can be calculated using the following formula: Ncontrol =  (*n* + 1) ∗ *P* (1 − *P*) ∗  ${\left(U_{\alpha }+U_{\beta }\right)}^{2}$/*n* (*P*1 − *P*2)^2^ (in this context, n represents the ratio of the control group to the case group. The variable P denotes the average exposure level. *U*_*α*_ is the critical value derived from the standard normal distribution, corresponding to the desired statistical power of the study. Similarly, *U*_*β*_ is the critical value from the standard normal distribution that aligns with the specified confidence level. The variables *P*1 and *P*2 indicate the exposure ratios between the disease group and the control group, respectively). According to the formula, the minimum sample size required for the case group was calculated to be 80 participants. Utilizing a 1:2 matching ratio, the minimum sample size for the control group was determined to be 160 participants.

**Figure 1:**
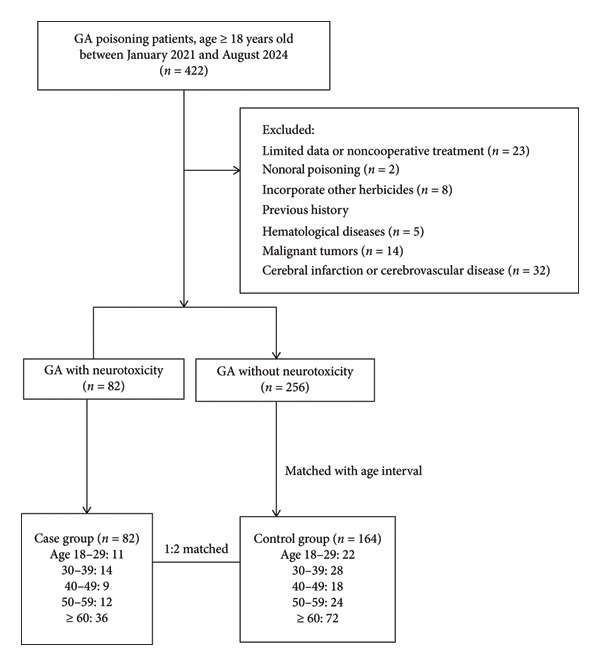
Flowchart illustrating the selection of the case group and the control group.

The inclusion criteria for the control group comprised patients diagnosed with GA poisoning, as defined by the diagnostic criteria specified in ICD‐10 code T60.3, confirmed through blood or urine toxicity testing, and who exhibited no signs of nervous system injury following toxic exposure. The inclusion criteria for participants in the case group comprised patients exhibiting characteristic symptoms of neurotoxicity—such as memory impairment, coma, convulsions and seizures, irritability, and disorientation—alongside imaging evidence of nervous system lesions subsequent to GA poisoning [15, 16]. The exclusion criteria for the control group and the case group were established to ensure the study’s validity and included several critical factors. Firstly, individuals under 18 years of age were excluded, given the significant differences in physiology and responses to poisoning between adults and minors. Additionally, patients who experienced nonoral routes of poisoning were not considered, with the focus placed solely on those who ingested the toxin orally. Participants who simultaneously exhibited signs of other pesticide poisoning, verified through blood or urine tests, were also excluded to prevent confounding variables that could affect the study’s outcomes. Moreover, any subjects with incomplete clinical data related to GA poisoning were eliminated from the control group. Patients with a pre‐existing history of neurological deficits or those presenting neurological symptoms before hospital admission were also excluded from the study. This exclusion criterion encompassed conditions such as hepatic encephalopathy, renal encephalopathy, pulmonary encephalopathy, metabolic encephalopathy, primary neurological disorders, and cerebral trauma. A total of 422 patients with GA poisoning were initially enrolled in the study. Following the application of specific inclusion and exclusion criteria, individuals were excluded for the following reasons: insufficient data or noncompliance with treatment (23 patients), concurrent use of other herbicides (8 patients), nonoral routes of poisoning (2 patients), a prior history of hematological disorders (5 patients), malignant tumors (14 patients), and a history of cerebral infarction or cerebrovascular disease before presentation at the emergency department (32 patients). Ultimately, 82 patients who developed neurotoxicity subsequent to GA poisoning were assigned to the case group. The control group was selected from the remaining GA poisoning patients who did not exhibit neurotoxicity, at a ratio of 1:2 (case group to control group). Controls were matched with cases based on age, categorized into 10‐year intervals (18–29 years, 30–39 years, 40–49 years, 50–59 years, and ≥ 60 years) (Figure 1).

### 2.2. Detection Methods

Clinical data were retrospectively collected and reviewed from the HIS system (China Anxiang Wisdom System 3.0). The following parameters were collected: age, gender, body mass index (BMI), the incidence of gastric lavage and hemodialysis procedures, initial Glasgow Coma Scale (GCS) score at admission, plasma GA concentration, medical history, and laboratory tests (blood routine examination, liver enzymes, kidney function, markers of myocardial injury, lactic acid, and initial serum ammonia); the levels of Lp‐PLA2 were measured using enzyme‐linked immunosorbent assay (ELISA) techniques, and the SII was calculated based on the formula involving peripheral blood cell counts: (neutrophil count ∗ platelet count)/lymphocyte count. All laboratory tests were ordered, and blood samples were taken within 24 h of admission. This study was approved by the Ethics Committee of Jinling Hospital, Medical School of Nanjing University. All methods were performed in accordance with the Declaration of Helsinki. All patients or guardians gave informed consent to the study.

### 2.3. Statistical Analysis

Statistical analysis was performed using IBM SPSS (version 24). Continuous variables were clarified by the distribution type according to the Shapiro–Wilk normality test. Normally distributed data were presented as mean and standard deviation, and non‐normally distributed data were presented as median with interquartile range. The Mann–Whitney U‐test and independent‐samples t‐test were used in comparing the continuous variables, respectively. Categorical variables were expressed as frequencies or percentages, which were compared by the chi‐square or Fisher’s exact tests. Logistic regression analysis was performed for single‐factor indicators with statistical significance. The area under the curve (AUC) and the diagnostic value of the receiver operator characteristic (ROC) curve were compared to the best indicators. The optimal cutoff points of predictors and 95% confidence intervals (95% CIs) were calculated. *p* < 0.05 was considered statistically significant.

## 3. Results

A total of 82 acute oral GA poisoning patients with neurotoxicity were identified as the case group along with 164 GA poisoning patients without neurotoxicity matched as the control group. Of the 246 sampled subjects, the median age was 56.0 years (35.0, 73.0). The median age for the case group and the control group was 55.0 and 56.0 years, respectively (*p* = 0.806). After performing age stratification and age matching, no statistically significant age discrepancies were observed between the case and control groups. As indicated in Table 1, there was no statistically significant difference between the two groups in terms of gender, hypertension, diabetes mellitus, chronic heart disease, initial GCS score, BMI, and the proportion of patients undergoing gastric or hemodialysis (*p* > 0.05). In addition, we analyzed the laboratory index between the case and control groups. The results showed that the levels of plasma GA concentration, SII, NLR, Lac, serum ammonia, and Lp‐PLA2 were statistically higher in the case group than in the control group (*p* < 0.05) (Table 2).

**Table 1:** Baseline characteristics of GA patients in the case group and the control group.

Variable	Case group (*n* = 82)	Control group (*n* = 164)	*z*/chi‐square value	*p*‐Value
Age (years)			−0.246^∗^	0.806
18–29	11	22		
30–39	14	28		
40–49	9	18		
50–59	12	24		
≥ 60	36	72		
Gender			2.088^∗^	0.148
Male, *n* (%)	38 (46.3%)	72 (43.9%)		
Female, *n* (%)	44 (53.7%)	92 (56.1%)		
Medical history				
Hypertension, *n* (%)	16 (19.5%)	23 (14.0%)	1.234^#^	0.267
Diabetes mellitus, *n* (%)	12 (14.6%)	17 (10.4%)	0.958^#^	0.328
Chronic heart disease, *n* (%)	6 (7.3%)	18 (11.0%)	0.831^#^	0.362
Initial GCS	12.0 (11.0, 14.0)	12.0 (11.0, 14.0)	−1.097^#^	0.273
BMI	22.6 (20.1, 25.0)	23.0 (18.0, 26.0)	−0.404^#^	0.686
Gastric lavage, *n* (%)	78 (95.1%)	160 (97.6%)	1.034^#^	0.309
Hemodialysis, *n* (%)	71 (86.6%)	147 (89.6%)	0.504^#^	0.478

Abbreviations: BMI, body mass index; Initial GCS, initial Glasgow Coma Scale.

^∗^Chi‐square test.

^#^Mann–Whitney *U*‐test.

**Table 2:** Laboratory tests of GA patients in the case group and control group.

Variables	Case group (*n* = 82)	Control group (*n* = 164)	*t/z*	*p*‐Value
Plasma GA concentration (mg/L)	3.3 (2.6, 3.8)	2.7 (2.1, 3.5)	−3.653^#^	< 0.001
SII (∗ 10^11^/L)	411.3 (289.7, 512.4)	253.9 (200.7, 334.5)	−7.188^#^	< 0.001
NLR	24.5 (18.7, 31.1)	20.0 (15.7, 22.9)	−5.143^#^	< 0.001
Scr (μmol/L)	136.9 (87.3, 182.7)	124.7 (94.9, 178.3)	−0.114^#^	0.909
Bun (mmol/L)	9.9 (8.3, 11.3)	9.5 (7.9, 11.1)	−1.140^#^	0.254
CK‐MB (U/L)	27.0 (21.8, 33.0)	26.5 (21.0, 33.0)	−0.230^#^	0.818
TnI (ng/mL)	0.01 (0.01, 0.03)	0.01 (0.01, 0.02)	−1.891^#^	0.059
AST (U/L)	41.0 ± 7.4	39.8 ± 10.7	−1.067^Δ^	0.287
ALT (U/L)	40.0 (29.0, 48.3)	36.0 (30.0, 43.0)	−1.682^#^	0.093
Lac (mmol/L)	2.8 (2.1, 3.4)	2.4 (1.7, 3.2)	−2.339^#^	0.019
Serum ammonia (μmol/L)	57.5 (50.0, 71.4)	54.1 (42.2, 60.8)	−3.630^#^	< 0.001
Lp‐PLA2 (ng/mL)	196.0 (167.8, 241.8)	124.0 (89.3, 164.5)	−9.725^#^	< 0.001

*Note:* Scr; serum creatinine; TnI, troponin I; AST, aspartate transaminase; ALT, alanine aminotransferase; Lac, lactate; Lp‐PLA2, lipoprotein‐associated phospholipase A2.

Abbreviations: Bun, blood urea nitrogen; CK‐MB, creatinine kinase‐MB; NLR, neutrophil‐to‐lymphocyte ratio; SII, Systemic‐Immune Inflammatory Index.

^#^Mann–Whitney *U*‐test.

^Δ^ *t*‐test.

**Figure 2:**
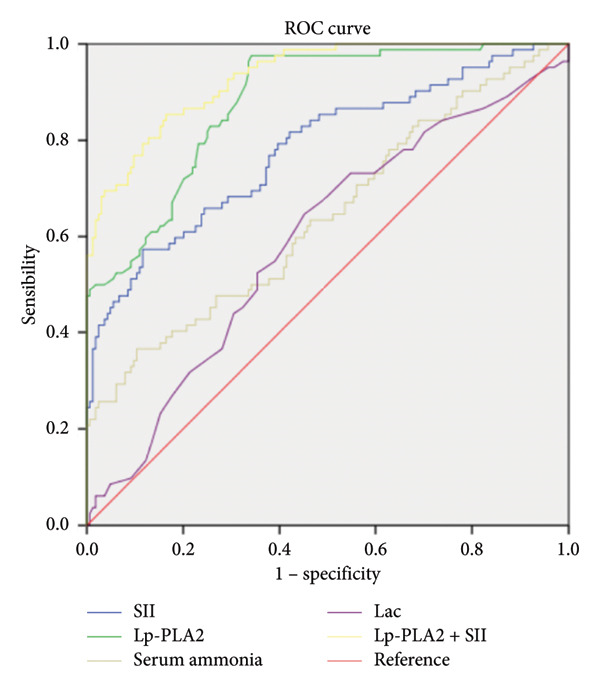
Receiver operating characteristic curves of SII, Lp‐PLA2, serum ammonia, Lac, Lp‐PLA2, and SII combination. Abbreviations: SII, systemic inflammatory index; Lp‐PLA2, lipoprotein‐associated phospholipase A2; Lac, lactate.

Multiple logistic regression analyses were performed to identify factors related to neurotoxicity associated with GA poisoning. The variables with statistical significance in the univariate logistic regression were incorporated into the multiple logistic regression analyses. As shown in Table 3, multiple logistic regression analysis showed that increased levels of SII, Lac, serum ammonia, and Lp‐PLA2 had ORs of the case group of 1.010 (95% CI: 1.004, 1.015), 1.656 (95% CI: 1.031, 2.660), 1.054 (95% CI: 1.017, 1.092), and 1.049 (95% CI: 1.032, 1.065) compared with the control group, respectively. The ROC curve and AUC were performed to assess the optimal predictors and the predictive performance of each individual predictor for the neurotoxicity caused by GA poisoning. The results showed that AUCs of SII, Lac, serum ammonia, Lp‐PLA2, and combination of Lp‐PLA2 with SII were 0.781 (95% CI: 0.717, 0.845, *p* < 0.001, cutoff = 394.6 ∗ 10^11^/L, with sensitivity of 57.3% and specificity of 88.4%), 0.591 (95% CI: 0.516, 0.667, *p* = 0.019, cutoff = 394.6 ∗ 10^11^/L, with sensitivity of 64.6% and specificity of 54.9%), 0.642 (95% CI: 0.566, 0.718, *p* < 0.001, cutoff = 64.2 μmmol/L, with sensitivity of 36.6% and specificity of 89.6%), 0.880 (95% CI: 0.838, 0.923, *p* < 0.001, cutoff = 150 ng/mL, with sensitivity of 97.6% and specificity of 65.9%), and 0.931 (95% CI: 0.901, 0.962, *p* < 0.001, with sensitivity of 85.4% and specificity of 83.5%), respectively (Table 4 and Figure 2).

**Table 3:** Univariate and multiple logistic regression analyses for the predictor of neurotoxicity induced by GA poisoning.

Variables	Crude OR (95% CI)	Adjusted OR (95% CI)	*p*‐Value (adjusted OR)
Plasma GA concentration	1.667 (1.241, 2.240)	1.534 (0.977, 2.410)	0.063
SII (∗ 10^11^/L)	1.010 (1.007, 1.013)	1.010 (1.004, 1.015)	< 0.001
NLR	1.166 (1.107, 1.228)	1.014 (0.922, 1.116)	0.774
Lac (mmol/L)	1.387 (1.028, 1.871)	1.656 (1.031, 2.660)	0.037
Serum ammonia (μmol/L)	1.044 (1.024, 1.065)	1.054 (1.017, 1.092)	0.004
Lp‐PLA2 (ng/mL)	1.043 (1.031, 1.054)	1.049 (1.032, 1.065)	< 0.001

*Note:* Lac, lactate; Lp‐PLA2, lipoprotein‐associated phospholipase A2.

Abbreviations: 95% CI, 95% confidence interval; NLR, neutrophil‐to‐lymphocyte ratio; OR, odds ratio; SII, Systemic‐Immune Inflammatory Index.

**Table 4:** AUC analysis for the predictor of neurotoxicity induced by GA poisoning.

Variables	AUC	*p*	95% CI	Cutoff	Sensitivity (%)	Specificity (%)
Serum ammonia (μmol/L)	0.642	< 0.001	0.566, 0.718	64.2	36.6	89.6
Lac (mmol/L)	0.591	0.019	0.516, 0.667	2.5	64.6	54.9
SII (∗ 10^11^/L)	0.781	< 0.001	0.717, 0.845	394.6	57.3	88.4
Lp‐PLA2 (ng/mL)	0.880	< 0.001	0.838, 0.923	150.0	97.6	65.9
Lp‐PLA2 and SII combination	0.931	< 0.001	0.901, 0.962	—	85.4	83.5

*Note:* Lac, lactate; Lp‐PLA2, lipoprotein‐associated phospholipase A2.

Abbreviation: SII, Systemic‐Immune Inflammatory Index.

## 4. Discussion

Despite the perception that GA exhibits low toxicity to humans and the environment, it remains a significant health risk in our country due to its delayed symptom onset [17]. The primary mechanism of GA involves the inhibition of the glutamine synthetase enzyme, leading to elevated blood ammonia levels and subsequent damage to various organs, particularly the nervous system, resulting in tissue necrosis [18, 19]. Glutamine synthetase is an essential enzyme in the human body, playing a critical role in brain metabolism and the normal functioning of various organs [20]. The hyperammonemia induced by herbicides is challenging to mitigate with lactulose, contrasting with the elevated serum ammonia levels observed in hepatic encephalopathy [21]. Early detection and intervention of GA neurotoxicity are vital for improving the prognosis of GA poisoning. Many patients with GA poisoning initially present without obvious symptoms but develop neurotoxic complications during treatment, posing significant challenges for emergency physicians. Currently, there are no reliable and easily detectable indicators for the early identification of GA neurotoxicity.

This study sought to identify risk factors for predicting brain injury associated with GA poisoning. Our results corroborate previous research indicating that serum ammonia concentration may be a predictor of GA‐induced neurotoxicity [9]. Importantly, the elevation in serum ammonia levels among patients experiencing neurotoxicity due to GA exposure was statistically significant compared to those without brain injuries. However, despite this observed correlation, the ROC curve was relatively modest at 0.642, and the sensitivity was only 36.6%, reflecting poor sensitivity and limited predictive accuracy in our research. Consequently, although serum ammonia levels provide some insight into the potential neurotoxicity associated with GA poisoning, their utility as an independent predictive marker in clinical practice is limited. Previous studies have highlighted the significant role of CSF ammonia levels in predicting neurological outcomes in GA poisoning, suggesting that they may serve as an indicator of nerve injury [10]. The challenge of obtaining CSF ammonia concentrations further complicates their clinical utility. Beyond serum ammonia levels, previous studies have explored additional factors. Notably, the GCS and age have been suggested as indicators of GA‐induced brain damage [5, 22]. Interestingly, our study did not reveal any statistically significant differences in GCS scores between the case and control groups. This outcome may be attributed to the timing of the GCS assessment, which was performed upon admission to the emergency department rather than during the period of neurotoxicity. The GCS is a dynamic instrument that assesses a patient’s neurological status at a specific moment, and it may not sufficiently detect the subtle changes in a patient’s condition immediately following GA exposure [23, 24].

This is the first epidemiological study conducted assessing the association between SII, Lp‐PLA2, and neurotoxicity caused by GA poisoning. SII is a novel biomarker reflecting immune status and vascular inflammation, which is readily measurable and has been identified as an independent risk factor for acute ischemic stroke and cardiovascular events [25, 26]. The role of Lp‐PLA2 has been extensively studied, particularly in relation to cerebrovascular disease, including ischemic stroke and cerebral infarction. Its levels may serve as a potential biomarker for predicting cerebrovascular diseases [27]. Nonetheless, the potential of Lp‐PLA2 and SII to predict neurotoxicity associated with GA toxicity remains to be elucidated. As a result of our findings, we noted the particular vulnerability of elderly patients to present with neurotoxicity following GA exposure. Recognizing the age‐related increased susceptibility is vital for understanding the appropriateness of specific risk factors and adjustments made during statistical analyses. To address this confounding factor, we implemented an age‐matching protocol in our case–control study design. This methodological adjustment helped to elucidate the potential associations more accurately, as older patients tend to present with different physiological and cognitive baselines that could skew predictive assessments. In the current study, a 1:2 matching ratio was implemented for the control group based on the age stratification intervals of the case group. Our findings revealed a significant increase in the expression levels of both SII and Lp‐PLA2 in the case group, further indicating their relevance as independent risk factors for predicting neurotoxicity associated with GA poisoning. Multiple logistic regression analyses indicated that SII (OR = 1.010, 95% CI: 1.004–1.015, *p* < 0.001) and Lp‐PLA2 (OR = 1.049, 95% CI: 1.032–1.065, *p* < 0.001) were independent risk factors for predicting the neurotoxicity induced by GA poisoning after adjusting for the levels of serum ammonia, Lac, and GA concentration. Our study also employed ROC curve analysis to evaluate the diagnostic performance of SII and Lp‐PLA2. The AUC for SII was 0.781, indicating a good predictive ability, with a cutoff value of 394.6 ∗ 10^11^/L. At this threshold, the sensitivity reached 57.3%, whereas the specificity was relatively high at 88.4%. This suggested that SII may play a role in identifying patients at risk for neurotoxicity associated with GA poisoning, although its moderate sensitivity indicated that it alone may not detect all at‐risk patients. Furthermore, Lp‐PLA2 demonstrated a higher AUC of 0.880, with a cutoff value of 150 ng/mL, resulting in an impressive sensitivity of 97.6% but a lower specificity at 65.9%. Importantly, when SII and Lp‐PLA2 were assessed in combination, the predictive performance improved considerably, yielding an AUC of 0.931. The sensitivity increased to 85.4%, and specificity was maintained at a robust 83.5%. These findings emphasized the clinical utility of these biomarkers and suggested that they could potentially be integrated into routine clinical practice for the management of GA poisoning patients with neurotoxicity.

The precise mechanisms responsible for the increased levels of SII and Lp‐PLA2 following exposure to GA and the subsequent nerve injury remain incompletely understood. A pivotal factor in this process appears to be the detrimental impact of GA on endothelial cells within the brain, which are integral components of the blood–brain barrier (BBB). The integrity of the BBB is crucial for preserving the homeostasis of the central nervous system’s microenvironment, and any disruption to this barrier can precipitate significant neurological consequences [28]. It has been suggested that GA exerts its neurotoxic effects by impairing endothelial cell function. Damage to these cells may lead to increased permeability, thereby permitting the infiltration of inflammatory mediators and other substances into the brain. The infiltration can subsequently initiate a cascade of inflammatory responses, thereby exacerbating neuronal injury and contributing to the observed increases in SII and Lp‐PLA2. Furthermore, elevated levels of SII and Lp‐PLA2 not only indicate ongoing inflammation but may also serve as potential predictors of neurodegenerative processes following GA exposure. The presence of these markers may reflect the extent of neuroinflammation and the degree of endothelial injury, positioning them as critical indicators for assessing the overall impact of GA toxicity on neuronal damage.

## 5. Conclusions

Our study highlighted the critical role of SII and Lp‐PLA2 as independent risk factors for predicting neurotoxicity in cases of GA poisoning. The promising predictive capability of these markers, especially when analyzed in conjunction, underscores their potential utility in clinical settings.

## 6. Limitations

It is essential to acknowledge the limitations inherent in our study. The retrospective cohort study design is susceptible to biases related to data collection and patient selection. In particular, the exclusion of certain control patients during the age‐stratified matching process may introduce potential bias. Future research should focus on prospective studies involving larger and more diverse populations to validate our results and enhance the robustness of our conclusions. Such studies should aim to elucidate the causative mechanisms underlying the observed associations and confirm the reliability of Lp‐PLA2 and SII as biomarkers in larger cohorts. Longitudinal studies examining temporal changes in these biomarkers in relation to glufosinate exposure would further clarify their prognostic value. Additionally, research into potential therapeutic interventions targeting the inflammatory pathways associated with Lp‐PLA2 could offer new strategies for mitigating neurotoxic damage from agricultural chemicals such as GA.

## Ethics Statement

This study was approved by the Ethics Committee of Jinling Hospital, Medical School of Nanjing University (Approved ID: 2021DZSKT‐YBB‐014). All patients or guardians gave informed consent to the study.

## Consent

The authors have nothing to report.

## Conflicts of Interest

The authors declare no conflicts of interest.

## Author Contributions

Xiang Xue and Xinyao Wu contributed equally to this work.

## Funding

This study was supported by Major project of Scientific Research Program for Colleges and Universities of Anhui Provincial Education Department (2022AH040168); Key Programs of Wannan Medical College (WK2021ZF04).

## Data Availability

The datasets used and analyzed during the current study are available from the corresponding author on reasonable request.
